# Noninvasive Ventilation Effectiveness in Amyotrophic Lateral Sclerosis

**DOI:** 10.3390/jcm14238609

**Published:** 2025-12-04

**Authors:** Jesús Sancho, Santos Ferrer, Jaime Signes-Costa

**Affiliations:** 1 Respiratory Medicine Department, Hospital Clinico Universitario, 46010 Valencia, Spain; santosfeesp@gmail.com (S.F.); jaimesignescosta@gmail.com (J.S.-C.); 2 Institute of Health Research INCLIVA, 46010 Valencia, Spain; 3 Medicine Department, Medicine Faculty, Universidad de Valencia, 46010 Valencia, Spain; 4 ALS Reference Unit of Comunidad Valenciana, 46010 Valencia, Spain

**Keywords:** amyotrophic lateral sclerosis, noninvasive ventilation, survival, upper airway obstruction

## Abstract

Amyotrophic lateral sclerosis (ALS) is a progressive neurodegenerative disease that affects motor neurons; respiratory problems are the leading cause of death and hospital admissions and are secondary to progressive weakness of the respiratory muscles and upper airway. Noninvasive ventilation (NIV) can increase survival, alleviate symptoms, reduce hospital admissions, and improve the quality of life of these patients. The key factor in respiratory management of patients with ALS is achieving effective NIV; ineffective NIV has a negative impact on survival, with a reduction of up to 50% compared to patients with an effective technique. The most common cause of ineffective NIV is air leaks; other causes include upper airway obstruction events, residual hypoventilation, hyperventilation, and upper airway obstruction secondary to an oronasal mask. Regular monitoring of the effectiveness of NIV is essential given its impact on survival; the key tools that detect the main problems are the presence of hypoventilation symptoms, arterial blood gases, nocturnal oximetry and capnography, and built-in ventilator software. Different measures have been proposed to address the ineffectiveness of NIV, such as fitting the mask to reduce air leaks, increasing ventilatory support for residual hypoventilation, decreasing ventilatory support for hyperventilation, or a trial with a nasal mask to address oronasal interface effects. In the case of obstruction, the most common measure is to increase positive expiratory pressure during NIV. These measures enable NIV to be effective in 58% of cases, achieving a survival rate similar to that of patients who have effective NIV from the outset.

## 1. Introduction

**Figure 1:**
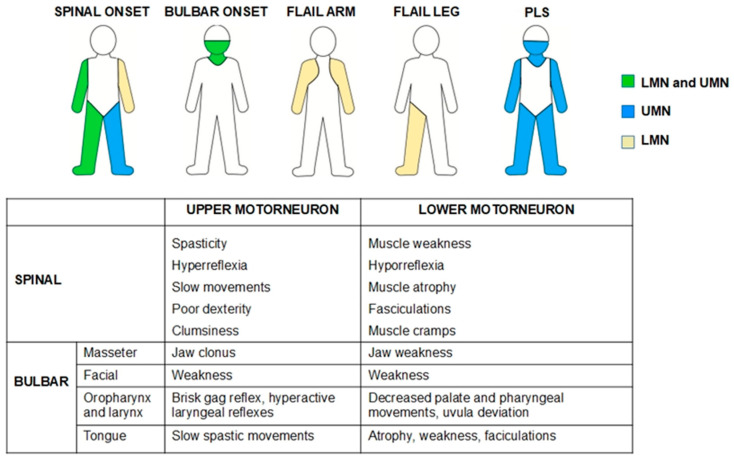
The most common phenotypes in ALS patients and signs of upper- and lower-motor-neuron involvement. LMN—Lower motor neuron, PLS—Primary Lateral Sclerosis, UMN—Upper motor neuron.

Amyotrophic lateral sclerosis (ALS) is a progressive neurodegenerative disorder that affects motor neurons, upper, lower, or both, causing progressive muscle weakness. Depending on the type of symptoms at the onset of the disease, ALS is classified as spinal, bulbar, or respiratory (Figure 1). However, regardless of the type of onset, around 80% of patients will eventually develop bulbar symptoms during the course of the disease (dysarthria, swallowing disorders, sialorrhea, etc.) [1]. In 10–15% of cases, an additional diagnosis of frontotemporal dementia (FTD) may be made, and between 35 and 40% of patients will experience mild behavioral and/or cognitive changes [2].

Although the cause of this disorder is unknown, different mechanisms have been implicated in its pathogenesis, such as protein homeostasis imbalance in neurons, RNA metabolism alteration, mitochondrial dysfunction, and the accumulation of protein aggregates in the cytoplasm of neurons with a toxic gain of function. In 85% of cases, they occur sporadically with an idiopathic etiology; several environmental factors have been implicated in the development of the disease, such as smoking, physical activity, and exposure to metals, pesticides, and electromagnetic fields [3]. In 15% of patients, a genetic cause is described. In these familial cases, the most common pathogenic variants are hexanucleotide expansions in open reading frame 72 of chromosome 9 (C9orf72) and mutations in superoxide dismutase 1 (SOD1), TAR DNA-binding protein 43 (TARDBP), fusion protein in sarcoma (FUS), and TANK-binding kinase 1 (TBK1) [3].

Without specific respiratory treatment, survival is 3 to 5 years after the onset of the disease, with respiratory problems being the leading cause of death and the main reason for hospital admissions [4,5]. Respiratory problems are the result of degeneration of the respiratory and upper airways motor neurons, leading to progressive respiratory failure and decreased cough effectiveness. The progressive degeneration of the phrenic motor neurons causes progressive weakness of the diaphragmatic muscle. This diaphragmatic dysfunction initially affects the rapid eye movement (REM) sleep phase, in which the accessory respiratory muscles are more relaxed, there is hypotonia of the upper airway, and there is a decrease in respiratory drive. Subsequently, due to the progression of the disease, it will spread to affect all phases of sleep. Respiratory muscle aids, noninvasive ventilation (NIV), and assisted coughing techniques have been shown to increase survival, improve symptoms and quality of life, and reduce hospitalizations in patients with ALS [6,7].

This narrative review aims to provide an overview of current knowledge on the efficacy of NIV in patients with ALS. Therefore, the objectives of this review are as follows: (i) to emphasise the importance of achieving effective NIV in ALS patients; (ii) to review the causes of ineffective NIV in these patients; and (iii) to describe the current tools for detecting ineffective NIV and the proposed solutions. To provide a broad representation of the available literature, we conducted a non-systematic literature search using PubMed, Embase, and Web of Science to identify relevant published review articles, clinical studies, and case reports focusing on the efficacy of NIV in ALS.

## 2. Noninvasive Ventilation and ALS Survival

Several studies have been published showing that NIV in patients with ALS increases survival compared to ALS patients who have not undergone NIV or who receive conservative therapy [7,8,9,10,11,12,13]. In cases where NIV is not tolerated, accepted, or effective, tracheostomy is the other life-sustaining alternative. Tracheostomy in patients with ALS allows for adequate alveolar ventilation and secretion management by bypassing the upper airway and directly accessing the tracheobronchial tree. However, it will not prevent the disease from progressing and involves a higher level of care for the patient at home and greater complexity, in most cases, provided by family members. At the same time, it should be noted that many patients reject this procedure. Therefore, survival in ALS patients on NIV is considered a tracheostomy-free period.

Bourke SC et al. published the only randomized controlled trial on NIV in patients with ALS [7]. This study, with some limitations (not using mechanically assisted coughing techniques and including patients with NIV use of less than 4 h), concluded that NIV significantly increased survival compared to ALS patients who did not use it [7]. Other non-randomized studies that used patients who refused NIV as controls obtained the same results, showing increased survival in patients who received NIV. The median reported survival from ALS onset in patients who used NIV ranges from 21 to 48 months; in those who did not use NIV, the reported survival ranges from 8 to 15 months [14].

ALS is a progressive disease, so muscle weakness will worsen over time, including weakness of the respiratory muscles. In this regard, weak respiratory muscles will not only be unable to maintain adequate ventilation during sleep, but also while awake. At that point, it will be necessary to increase the hours of NIV use beyond sleep and to modify ventilation parameters, especially if pressure-cycle ventilation modes are used [11,15].

The main prognostic factor for ALS patients who have started NIV is the severity of bulbar dysfunction [11,16,17]. The median survival in patients who started NIV with moderate bulbar dysfunction or no bulbar dysfunction was 20 months, and in those with severe bulbar dysfunction, 13 months [16]. Other prognostic factors described are the onset of bulbar dysfunction [13,14], failure to control respiratory secretions [12], age, and the severity of ALS at the time of initiating NIV, as measured by the *revised amyotrophic lateral sclerosis functional scale* [12,18].

## 3. NIV Effectiveness in ALS

In addition to the prognostic factors described above, two factors determine the success of NIV in patients with ALS: tolerance and efficacy. Thus, in relation to NIV tolerance, it has been observed that patients who use NIV for more than 4 consecutive hours during the night have better survival than those who use it for less than 4 consecutive hours (22 vs. 15 months) [19]. Studies focusing on NIV tolerance in subjects with ALS have reported tolerance levels ranging from 46% to 90%. Various factors have been associated with lower NIV tolerance in ALS (<4 h/day) [19,20,21].

The severity of bulbar involvement in ALS patients at the time of initiating ventilation is the most important factor related to NIV tolerance [20,21]. Furthermore, NIV tolerance is six times more likely in patients with spinal-onset ALS than in those with bulbar-onset ALS. Bulbar dysfunction is associated with more difficult secretion control and drooling, which could interfere with the efficacy and adherence to NIV treatment; therefore, sialorrhea and ineffective control of respiratory secretions related to bulbar dysfunction have been associated with poor compliance with NIV [12,22]. The absence of respiratory symptoms at the start of NIV has been shown to be another negative predictor of adaptation and tolerance to NIV [22].

Neurocognitive impairment is present in up to 50% of patients with ALS [23,24] and may influence the efficacy of supportive therapies such as NIV. Chio A. et al. [25] concluded that different types of cognitive impairment significantly and negatively affect tolerance to NIV and, therefore, its efficacy. More recently, Russo M. et al. [22] reached similar conclusions. According to these authors, patients with neurocognitive impairment may show decreased motivation, reduced mental flexibility, impaired planning ability, and deficits in both expression and comprehension. This, combined with a poor understanding of the disease, may prevent them from appreciating the need for and importance of NIV, resulting in lower tolerance. In addition, depression may interfere with NIV adherence in patients with ALS.

**Figure 2:**
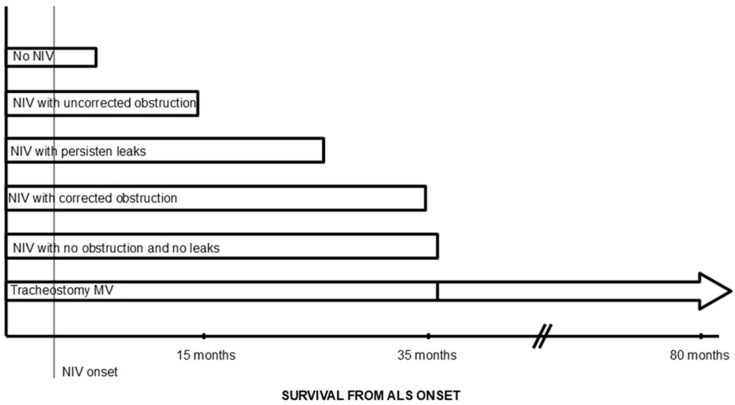
Diagram of expected survival in patients with ALS based on the efficacy of NIV. Modified from Figure 1 in reference [27]. Survival rates taken from references [9,12,16,20,26,28].

The two main factors determining the effectiveness of NIV in ALS patients are the presence of air leaks and the presence of obstructive events during NIV in the upper airway. González-Bermejo et al. observed that survival was lower in patients undergoing NIV with nocturnal desaturations, despite improvements in daily symptoms and daytime arterial blood gas values [26]. In 26% of patients, these desaturations were related to upper airway obstruction episodes during NIV. Subsequently, these same authors conducted a study to assess the role of these obstruction episodes in NIV failure in patients with ALS. The results of the study show that, after correction of leaks, around 49% of patients with ALS experienced obstructive events with NIV, although in one group of patients there were no desaturations; patients in whom obstructive events could not be corrected had lower survival rates compared with those with effective NIV (14 months vs. 30 months) [9]; even those with uncorrected obstructions and no desaturations had a worse prognosis (Figure 2).

In addition, frequent asynchronies between the patient and the ventilator have been described in patients with ALS during nocturnal NIV, with ineffective triggering being the most common asynchrony detected in patients with ALS [29,30]. This can compromise the effectiveness of NIV and negatively influence clinical outcomes. Ineffectiveness of NIV due to bulbar involvement has been described as the main cause of noninvasive treatment failure in patients with ALS, which has been reported in 49.38% of ALS patients using NIV in whom noninvasive treatment of respiratory problems fails [9,16]. Other reported causes of noninvasive treatment failure include failure to treat respiratory secretions and sudden death [31].

In light of these preliminary considerations, some authors have proposed that NIV is effective in ALS when used for more than 4 consecutive hours, the total sleep time with SpO_2_ below 90% while using NIV is <5%, the blood carbon dioxide pressure (PaCO_2_) is <45 mmHg, air leaks are corrected, the presence of obstructive events is <5/h, asynchronies occur <10% of the time recorded, and symptoms related to hypoventilation are alleviated [9,11,30].

## 4. Obstructive Events During NIV in ALS

Obstructive episodes during NIV are related to episodes of upper airway obstruction during NIV; these obstructive episodes have a negative impact on ALS survival and are one of the main causes of NIV failure in ALS [9]. These obstructive episodes have been reported to be present in 50 to 10 per cent of ALS patients receiving NIV [9,30]. Obstructive events are closely related to bulbar dysfunction and, in a recent study, have been shown to be more frequent in patients with predominant upper motor neuron dysfunction at the bulbar level [30]. This upper airway obstruction is associated with a decrease in respiratory neural drive, and Georges et al. found it predominantly occurred during REM sleep [9], whereas Sancho et al. found it occurred mainly during non-REM sleep [30]. This upper airway obstruction is associated with decreased respiratory neural drive, and Georges et al. found that it was predominantly during REM sleep [9], although Sancho et al. found that it was mainly during non-REM sleep [30]. It is important to consider that a percentage of ALS patients already show abnormalities during spontaneous breathing related to bulbar dysfunction at the upper airway, some of whom experienced worsening with NIV, and others develop new issues even if they initially had none [32]. From an anatomical point of view, obstruction was produced due to backward movement of a flaccid epiglottis (50%), paradoxical vocal fold movements (25%), soft palate collapse (33%), oropharyngeal collapse (25%), and retroglossal collapse (25%) [9,32].

### Causes of Obstructive Events During NIV in ALS

A number of ALS-related factors can theoretically increase upper airway obstruction related to bulbar dysfunction, due to both upper and lower motor neuron involvement at the bulbar level; bulbar dysfunction can produce weakness, hypotonia, or hyperreflexia at the upper airway. The decreased strength and hypotonia of the tongue, soft palate, pharynx walls and epiglottis would constitute a predisposing factor to increased upper airway collapsibility, even more in patients with no obvious bulbar lesion [33,34]. Retained oropharyngeal secretions and sialorrhea due to bulbar dysfunction may worsen the degree of obstruction. Moreover, degeneration of corticobulbar fibers with lesion of the upper motor neuron may cause hyperreflexia with exaggerated reflexes at the upper airway, such as glottic closure, inducing upper airway obstruction [34,35]. Some authors have proposed other mechanisms, described in different diseases, that may predispose to upper airway obstruction, such as chronic irritation of the surface of the pharynx, a higher propensity of the airways to collapse at low lung volume or upper airway nocturnal redistribution of interstitial fluid [9]. Another cause of upper airway obstruction during NIV is hyperventilation. Excessive ventilatory support decreases CO_2_, exceeding the apnoea threshold and inducing central apnoea with closure of the glottis to restore CO_2_ levels [36].

**Figure 3:**
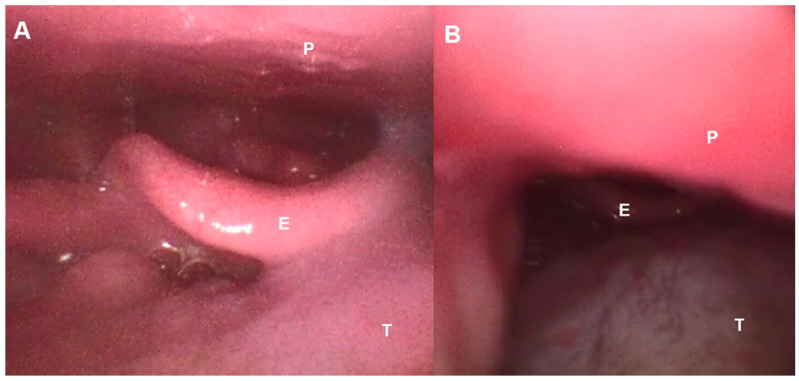
Fiberbronchoscopic image of the upper airway in an ALS patient. (**A**): Spontaneous breathing, (**B**): Under NIV with backward movement of tongue induced by oronasal mask. E—Epiglottis, P—Palate, T—Tongue.

On the other hand, as described in other pathologies, the use of oronasal masks during NIV can cause obstruction due to two mechanisms that decrease the anteroposterior diameter of the pharynx [36,37]: the mask and harness can cause compression and backward traction of the jaw, and the pressure generated by the ventilator can cause backward movement of the tongue and collapse of the epiglottis (Figure 3). On the other hand, it has been shown that mouth opening, induced by the oronasal mask or due to the weakness of the orofacial muscles produced in ALS, significantly reduces the retroglossal space and the anteroposterior diameter of the pharynx, which increases the collapsibility of the upper airways and even induces backward movement of the epiglottis.

Central factors have also been proposed as triggers of obstructive events during NIV in patients with ALS. Georges et al. [9] proposed that NIV could inhibit compensatory ventilation mechanisms; these authors argue that NIV can reduce PaCO_2_, which would decrease the overall ventilatory drive and could promote glottic closure. Furthermore, NIV could induce mechanical inhibition of the ventilatory drive, independent of any variation in CO_2_ and related to changes in thoracic afferents and their cortical processing [9]. Thus, some patients with ALS and respiratory failure may, in certain cases, show respiratory-related cortical activity during resting respiration, which is associated with more severe dyspnoea, and this cortical activity disappears under NIV [38]. The results of other studies show that there may be an alteration in ventilatory control in patients with ALS that could induce episodes of upper airway obstruction with decreased respiratory drive during NIV. Onders et al. [39] found that some ALS patients have minimal or no diaphragmatic activity as measured by electromyography, but show strong diaphragmatic contraction when stimulated via electrodes, suggesting a loss of upper motor neuron respiratory control. Similarly, Sancho et al. [30] observed that patients with upper airway obstruction during NIV had a predominance of upper motor neuron involvement at the bulbar level, greater controller gain, and lower CO_2_ reserve; furthermore, these episodes occurred in the non-REM phase and were accompanied by glottal closure. All these findings suggested instability in ventilation control in these patients and pointed to possible central involvement in this disease.

## 5. Detection of Non-Effective NIV in ALS

**Figure 4:**
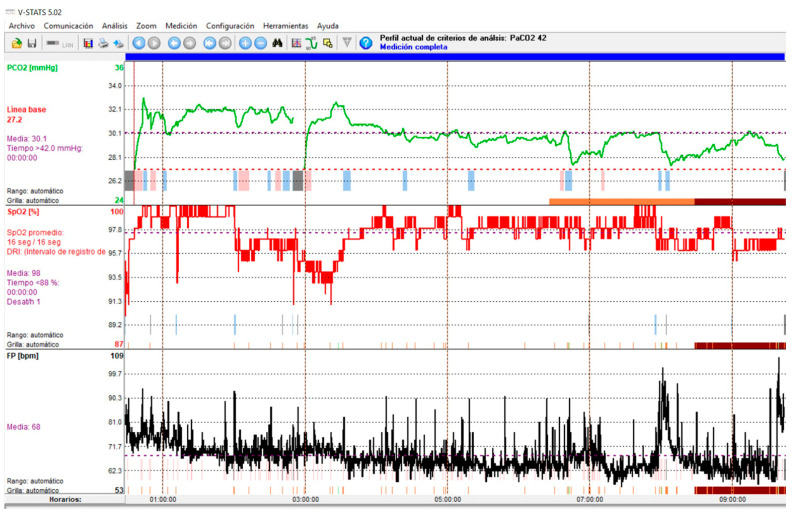
Transcutaneous capnography recording showing NIV-induced hyperventilation with hypocapnia. Green line: CO_2_; Red Line: SpO_2_; Black line: Heart rate.

Due to the consequences of ineffective NIV on survival in patients with ALS, regular monitoring of NIV efficacy is essential. Due to the pathophysiology of respiratory problems in ALS patients, which means that NIV is initially used during sleep, monitoring NIV during sleep may be preferable to daytime assessment. There are data that may suggest their presence, such as the persistence of hypoventilation symptoms, daytime hypercapnia, or elevated HCO_3_ levels despite NIV [40]. Periodic episodes of air leaks or the presence of aerophagia are factors associated with obstructive events; when the upper airway is obstructed, the air delivered by the ventilator will not reach the lungs and will either escape back outside, generating perimask air leaks, or be directed to the digestive tract, generating aerophagia. Periodic ventilator alarm triggers also suggest the presence of an ineffective NIV (disconnection alarm, low volume alarm for pressure ventilatory modes, high inspiratory pressure alarm for volume ventilatory modes, suggesting obstructive events; air leak alarms and low inspiratory airflow in pressure ventilatory modes or low peak inspiratory pressure alarms in volume ventilatory modes. Along with symptoms and arterial blood gas test results, nocturnal pulse oximetry is recommended [38]. A simple oximetric recording during NIV can provide information not only on residual oxygen desaturations, but also on sleep fragmentation (peripheral vasoconstrictor responses associated with micro-awakenings secondary to desaturations are visually identifiable by reductions in pulse wave amplitude that can be easily recorded using conventional pulse oximeters) [41]. The main limitation of the technique is the lack of specificity of SpO_2_ variations; thus, pulse oximetry tracings during NIV may show a wide variety of events; furthermore, normal SpO_2_ values do not rule out the presence of nocturnal hypercapnia in neuromuscular patients [42]. Most authors recommend adding a measurement of nighttime CO_2_. Of the different techniques for performing capnography (end-tidal/transcutaneous), transcutaneous is preferred because end-tidal CO_2_ measurement has significant limitations due to underlying lung disease and unintentional leaks during NIV, rendering the values unreliable [41] (Figure 4).

**Figure 5:**
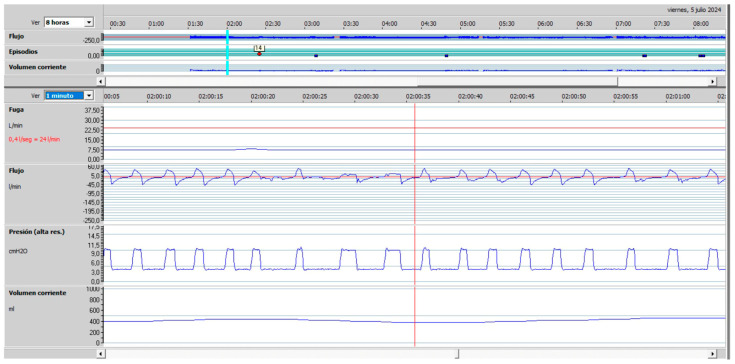
Upper airway obstruction with a decrease in inspiratory flow in a patient under pressure-cycled ventilation. (Red line Leaks, top blue line: Flow, bottom blue line: Pressure).

**Figure 6:**
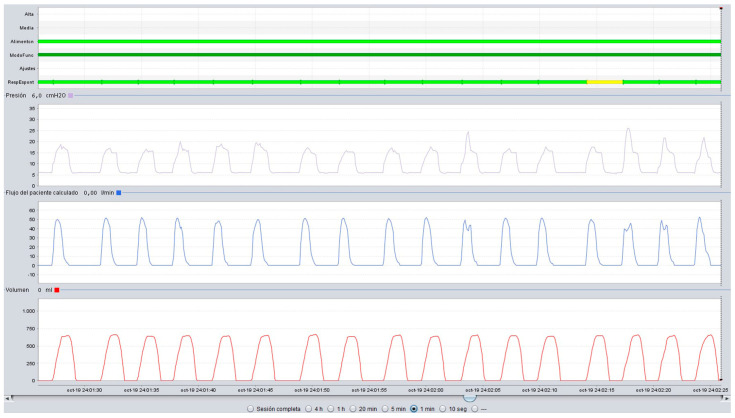
Upper airway obstruction with an increase in peak inspiratory pressure in a patient under volume-cycled ventilation. (Violet line: Pressure, Blue line: Flow, Red line: Volume).

In this regard, the presence of symptoms of hypoventilation, daytime hypercapnia (arterial blood gas analysis), nocturnal desaturations (pulse oximetry), or increases in PaCO_2_ during the night (nocturnal transcutaneous capnography) should point us towards ineffective NIV. Of the tools described above, some are basic and reflect the consequences of the interaction between the patient and the ventilator. Moreover, the alterations detected with these basic tools are non-specific and, apart from indicating that NIV is ineffective, they do not provide information about the possible cause [43]. Therefore, this basic monitoring must be supplemented with built-in software from the ventilator that is capable of recording a wide range of parameters over several months, and thus offering information on items such as compliance, leaks, tidal volume or respiratory rate. With this information, we can detect the presence of unintentional leaks, which are the most common cause of ineffective NIV. Furthermore, analysis of flow-time and pressure-time curves from built-in ventilator software will enable us to detect abnormal events during NIV and establish the causes of ineffectiveness, such as obstructive events, patient-ventilator asynchrony, or persistent hypoventilation [43]. In this regard, a decrease in inspiratory flow in pressure ventilation modes and an increase in peak inspiratory pressure in volume modes are indicative of obstruction (Figure 5 and Figure 6); an increase in inspiratory flow in pressure ventilation modes or a decrease in peak inspiratory pressure in volume modes are indicative of air leaks [43].

**Figure 7:**
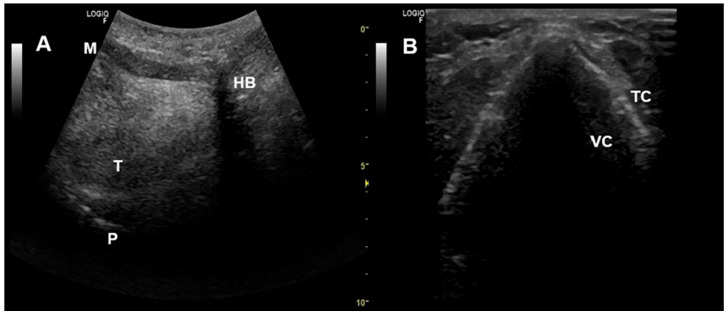
Upper airway ultrasound. (**A**) Oropharyngeal view. (**B**) Glottic view. HB—Hyoid bone, M—Mentum, P—Palate, T—Tongue, TC—Thyroid cartilage, VC—Vocal cords.

However, in complex cases or when the measures applied fail to correct the alterations and achieve an effective NIV, it is recommended to perform a polygraphy/polysomnography during NIV. A polygraph will show a decrease in respiratory drive (absence of thoracoabdominal movements), and a polysomnography allows us to assess the sleep phase in which the obstructions occur [43]. To identify the upper airway structures involved in the obstruction, which is important in obstructions secondary to an oronasal mask, imaging techniques during NIV and in spontaneous breathing, such as upper airway ultrasound (Figure 7) or nasofibroscopy (Figure 2), can be used [27,30,32,44].

## 6. Management of Ineffective NIV in ALS

Georges et al. found that ineffective NIV could be corrected in up to 58% of cases with no survival difference between patients corrected during treatment and those who were adequately ventilated at NIV onset [9]. The first step in optimising NIV in patients with ALS is to adequately control respiratory secretions and sialorrhea, as well as air leaks [9,12]. In cases where no obstruction is observed and persistent hypoventilation is detected, ventilatory support should be increased [27]. If symptoms of hypoventilation or daytime hypercapnia persist with effective NIV during the night, this indicates that the number of hours of NIV use should be increased; in this case, it is important to alternate between different types of masks (nasal or oral masks are recommended for daytime use) to prevent the development of ulcers and facilitate social interaction [15]. If the obstruction is secondary to hyperventilation, the solution is to reduce ventilatory support. In cases where the obstruction is related to the oronasal mask, several authors suggest switching to a nasal mask with or without a chin strap; however, air leaks through the mouth, aggravated by weak facial muscles, can make NIV ineffective or poorly tolerated by patients [36,37].

In general terms, the correction or improvement of obstructive events during NIV in ALS patients varies between 91% and 42% [9,32]. Different options have been proposed to solve these episodes. The most frequently effective proposed treatment is to increase expiratory positive airway pressure (EPAP) to high levels (from 4 to 14 cmH_2_O) in order to reduce upper airway collapsibility [9,27,40]. Unfortunately, this was not always effective and high levels of EPAP are not always tolerated; it has been found that increasing EPAP is effective in around 35% of ALS patients presenting obstructive events with NIV [9]. It should be noted that in one study, obstructive events were corrected in 36.7% of patients, and these episodes occurred during the REM phase of sleep [9].

However, in another study, increasing EPAP did not correct these events in any patients, and they occurred during the non-REM phase, and these patients had a predominance of upper motor neuron involvement at the bulbar level [30]. Perhaps the physiopathological mechanisms involved in the onset of obstructions in these two studies are different, hence the difference in response to increasing EPAP; in the first case, it could be a collapsability of the upper airway aggravated in the REM phase by increased hypotonia, and in the second case, an instability in control of ventilation, which in the non-REM phase depends mainly on chemoreceptors, and would cause a decrease in neural drive with glottis closure. Other authors have proposed the use of ventilatory modes with auto EPAP in order to improve upper airway patency with better tolerance, taking into account that automatic algorithms are different between the devices [27].

If increasing EPAP does not correct obstructive events, other proposed solutions include reducing inspiratory positive airway pressure (IPAP) and reducing inspiratory time. Finally, an alternative consists of switching from pressure-preset NIV to volume-preset NIV; in this regard, Sancho et al., using volume-preset NIV, found that only 10% of ALS patients presented obstructive episodes, and these events were localized at the glottic level [30].

Various authors have proposed the use of self-adapting inspiratory pressure ventilation in order to correct these obstructive events [41]; these ventilatory modes are adaptive ventilation modes that use an algorithm to adjust pressure support to target a specified tidal volume (volume-assure pressure support (VAPS)) or alveolar ventilation (intelligent VAPS (iVAPS)) [45,46,47]. However, various studies focusing on ALS patients have not found these modes to offer a clear benefit over conventional ventilation modes [45,46], and Panyarath et al. recently observed that patients with iVAPS have a higher residual apnoea-hypopnoea index at 6 months and resulting in a greater upper airway instability [47].

Other anecdotal alternatives to improve upper airway obstruction, described but with poor tolerance, are the cervical collar and mandibular advancement device during NIV [48,49].

The above reflects the complexity of NIV in patients with ALS. Therefore, the multidisciplinary management of the patient should include the participation of a pulmonologist who is an expert in ventilation, as improved survival will only be achieved if effective NIV is obtained [50].

## 7. Future Directions

This narrative review shows that there are still unclear aspects regarding NIV in patients with ALS that need to be further investigated in order to improve patient survival. Future research should focus on elucidating the cause of obstructive events not caused by hyperventilation or the use of an oronasal mask. It is also necessary to identify patients who improve with an increase in EPAP to correct obstructions and determine the optimal treatment for patients in whom this action does not correct these events.

## 8. Conclusions

NIV increases survival, improves symptoms and quality of life and decreases hospitalizations in ALS patients. Achieving effective NIV is crucial to meeting these objectives, as ineffective NIV leads to a decrease of up to 50% in survival rates (14 months vs. 30 months). In this regard, the effectiveness of NIV should be monitored periodically. The basic tools that allow us to monitor and detect the causes of ineffective NIV are the presence of hypoventilation symptoms, arterial blood gas analysis, nocturnal pulse oximetry, nocturnal capnography, and built-in ventilator software. The main cause of ineffective NIV in patients with ALS is air leakage, followed by upper airway obstruction events. In cases of air leaks, this can be resolved by properly adjusting the mask; for obstructions not due to hyperventilation or the effect of the oronasal mask on the upper airway, increasing the EPAP can correct these events in up to 34% of patients. After correcting obstructive events, survival is comparable to that of patients with effective NIV without leaks or obstructions.

## Data Availability

No new data were created or analyzed in this study. Data sharing is not applicable to this article.
